# Synthesis, Characterization, and Study of the Bioactivity of Nanosized Fe(III) and Cr(III) Coordination Polymers Based on Cross‐Linked Sodium Alginate and Polyacrylic Acid Biopolymers

**DOI:** 10.1155/ijbm/3245959

**Published:** 2026-07-03

**Authors:** Maged S. Al-Fakeh, Maryam Aldoghaim, Munirah S. Alazmi, Yassine El-Ghoul, Ahmed B. M. Ibrahim

**Affiliations:** ^1^ Department of Chemistry, College of Science, Qassim University, Buraidah, 51452, Saudi Arabia, qu.edu.sa; ^2^ Faculty of Applied Sciences, Taiz University, Taiz, 12372, Yemen, taiz.edu.ye; ^3^ Department of Chemistry, College of Science, King Faisal University, Al-Ahsa, 31982, Saudi Arabia, kfu.edu.sa; ^4^ Textile Engineering Laboratory, University of Monastir, Monastir, 5019, Tunisia, um.rnu.tn; ^5^ Department of Chemistry, College of Science, Imam Mohammad Ibn Saud Islamic University (IMSIU), Riyadh, 11623, Saudi Arabia, imamu.edu.sa

**Keywords:** anticancer, antimicrobial, antioxidant, cross-linked Na-AG and PAA biopolymer

## Abstract

This study focuses on the preparation and properties of nanosized Fe(III) and Cr(III) coordination polymers using cross‐linked sodium alginate (AG) and polyacrylic acid (PAA) as biopolymer matrices. The cross‐linked ligand biopolymer was prepared, and its reactions with iron(III) chloride and chromium(III) chloride were performed. The obtained complexes were characterized using techniques, including elemental analysis, FT‐IR, and UV–Vis. spectroscopy, XRD, and SEM, which confirmed structural integrity, electronic transitions, and unique morphologies of them. The crystallite size of all nanostructured coordination polymers was in the range of 35–40 nm. Thermal analysis (thermogravimetry, derivative thermogravimetry, and differential thermal studies) has been used to study the thermal decomposition stages. All the synthesized coordination polymers were nonelectrolytes with magnetic moments ranging from 3.91 to 5.92 BM. The antimicrobial and antioxidant capabilities of the nanosized metal coordination polymers confirmed their potential to mitigate oxidative stress and related disorders and revealed excellent biocompatibility of the polymeric complexes. Furthermore, preliminary anticancer studies indicated that these Fe(III) and Cr(III) nanosized coordination polymers exhibit significant cytotoxic effects against breast MCF‐7 cancer cell lines and no toxicity against normal cell lines, suggesting their potential as drug delivery systems or therapeutic agents.

## 1. Introduction

**FIGURE 1:**
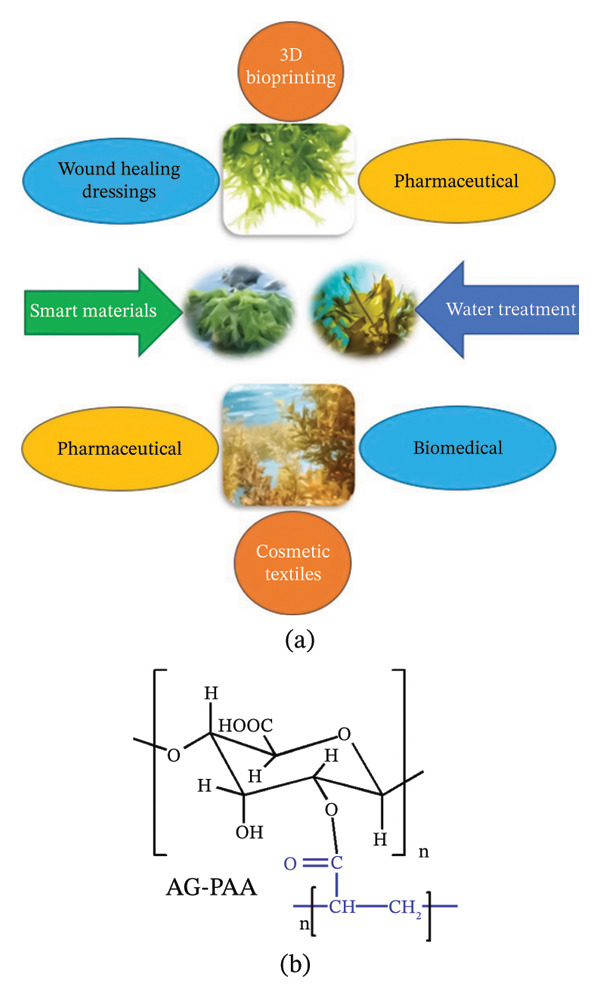
(a) Some applications of seaweeds. (b) Structure of the ligand.

Metal coordination polymers (MCPs) are a fascinating and versatile class of materials composed of metal ions coordinated to organic ligands, forming a three‐dimensional (3D) network. These materials, referred to as metal‐organic frameworks (MOFs) when the network structure is particularly porous, have garnered significant attention. The tunability of MCPs is one of the key factors driving their widespread interest and potential in diverse applications such as catalysis, drug delivery, and environmental remediation. The ability to modify the properties of MCPs by varying the metal ions, organic ligands, and synthesis conditions allows researchers to design materials with specific characteristics suited to these fields. Recent advancements in the field have focused on the synthesis of nanosized MCPs, particularly using biopolymers as the matrix to enhance the biocompatibility. Sodium alginate (AG) and polyacrylic acid (PAA) are two biopolymers that have shown great potential in forming stable networks for metal coordination. Sodium AG (SA), a naturally occurring biocompatible and biodegradable polysaccharide extracted from brown seaweeds, has excellent gel‐forming capabilities and is widely used in biomedical applications [1–5]. PAA, a synthetic polymer, provides a versatile platform for modifying properties such as pH sensitivity and adhesion [6]. The combination of these two biopolymers can create a robust and flexible matrix that supports the formation of nanosized MCPs. The synthesis of nanosized MCPs typically involves the coordination of metal ions (e.g., Cu^2+^, Cr^3+^, Fe^3+^, Zn^2+^, and Co^2+^) with functional groups present in the biopolymers. Cross‐linking of SA and PAA can be achieved through various methods, including ionic gelation or covalent cross‐linking, resulting in a porous structure that facilitates metal ion encapsulation. The choice of metal ions and the ratio of biopolymers can significantly influence the properties of the resulting MCPs, such as their surface area, porosity, and mechanical stability [7]. Due to their unique properties (e.g., higher surface area‐to‐volume ratio) and versatility, nanosized MCPs derived from cross‐linked SA and PAA have potential applications in several fields. In catalysis, these materials can serve as heterogeneous catalysts, improving reaction efficiency and recyclability. In drug delivery, the biocompatibility of the biopolymers allows for safe transport of therapeutic agents. In addition, these materials, which have high adsorption capacity and selectivity, can be employed in environmental applications (e.g., heavy metal ion removal from wastewater) [8–14]. Also, there are more applications in tissue engineering, wound healing, food industry, agriculture, cosmetics, and biomedical and environmental applications (Figure 1a) [15–19]. Previously, copper(II), nickel(II), and cobalt(II) AG biopolymer compounds were reported to show antibacterial, antifungal, and biosensing properties [20–23]. Fe(III) ions can participate in redox cycling and reactive oxygen species generation, which may enhance oxidative stress–mediated anticancer activity. In addition, chromium(III) complexes are known to exhibit strong coordination stability and potential biological activity through interactions with cellular biomolecules. Compared to previously reported Cu(II), Ni(II), and Co(II) complexes, Fe(III) and Cr(III) were expected to provide enhanced antioxidant and anticancer performance due to their distinct redox behavior, coordination properties, and ability to influence cellular oxidative pathways. Therefore, we propose that nanosized polymeric Fe(III) and Cr(III) complexes of PAA/AG biopolymer (Figure 1b) will show more antioxidant and anticancer activity. Here, we created the proposed complexes and studied their characteristics as well as their antifungal, antibacterial, antioxidant, and anticancer properties. The biological results estimated due to the complexes (especially the Fe(III) complex) are significant and can be used for the creation of drugs with a broad spectrum of activity against microbial and cancer cells. As well, the complexes appear to be safe against normal cells. Iron promotes oxidative stress–induced apoptosis in cancer cells by catalyzing ROS generation through the Fenton reaction. The resulting oxidative damage affects DNA, proteins, lipids, and mitochondria, leading to activation of apoptotic pathways and cell death. Iron is also involved in Ferroptosis, an iron‐dependent form of programmed cell death. A suitable rationale for selecting only Fe(III) and Cr(III) is that these ions are among the most environmentally relevant transition metals commonly detected in industrial wastewater, especially from mining, electroplating, steel manufacturing, and tannery effluents. Both metals exhibit strong coordination tendencies and well‐defined redox chemistry that directly influence adsorption, complexation, or catalytic behavior, making them ideal model ions for evaluating the performance of the synthesized material. Synthesis of eco‐friendly biopolymers of nanosized Iron(III) and Chromium(III) coordination complexes with AG/PAA biopolymers involves using biological agents like plants or microorganisms to create the nanoparticles, which are then combined with the biopolymer. This method is eco‐friendly and cost‐effective [24].

## 2. Experimental

### 2.1. Materials and Methods

This research involved Sigma‐Aldrich analytical‐grade materials (SA, PAA, methanol, dimethyl sulfoxide [DMSO], Fe(III) chloride hexahydrate, and Cr(III) chloride hexahydrate), which did not require purification. AG‐PAA was prepared according to procedures [24]. The GmbH Vario El analyzer determined all compounds’ elemental compositions. FT‐IR structural data were measured on a Thermo Nicolet (6700) spectrophotometer. Shimadzu (UV‐2101) PC spectrophotometer measured the UV–Vis spectra. The magnetic susceptibility balance “MSB‐Auto” was used to determine the magnetic moments, and conductance data were measured using a JENWAY model 4310 conductivity meter. An XRD diffractometer “PW 1710” utilizing Cu‐Kα radiation generated the powder diffraction patterns of the complexes (40 kV and 40 mA). The synthesized materials underwent morphological and structural examination through the use of a scanning electron microscope (ZEISS Ultra Plus).

### 2.2. Preparation of a Cross‐linked Polymeric Ligand (Poly‐PAA/AG)

We place 100 mL of distilled water in a round‐bottom flask and add 3 g of SA and vigorously stir for 1 h at 80°C. Then, we added dropwise a PAA solution that had been previously made by combining 3 g of PAA with 100 mL of H_2_O, stirring continuously, and heating at 70°C. Under N_2_ pressure, the mixture was stirred at 120°C for 2 hours. The reaction solution was then preserved at 90°C overnight while being stirred. The resultant suspension was then brought to 25°C before being concentrated at a lower pressure. The next step was to dry the product (90%) under high vacuum at 65°C to obtain a white color stable powder. The cross‐linked polymeric ligand (Poly‐PAA/AG) data are as follows: Anal. Calc. for C9H10O7: C, 46.97; H, 4.38. Found: C, 46.93; H, 4.98. IR data: *υ*(OH) 3222, *υ*(C‐H) 2922, *υ*(C=O) 1725, *υ*(COO) 1413, *υ*(CO) 1263, *υ*(C‐O‐C) 1025.

### 2.3. Preparation of Metal Complex Nanoparticles

AG‐PAA (0.78 g, 3.4 mmol) was dissolved in 40 mL of boiling methanol–water mixture (1:1). After the mixture had cooled, a solution of metal chloride hexahydrate (FeCl_3_.6H_2_O [0.94 g] or CrCl_3_.6H_2_O [0.91 g], 3.4 mmol) in 10 mL of distilled water was added. The precipitate of the chromium complex separated out on stirring after a few minutes, but refluxing of the iron‐containing solution resulted in precipitation of the solid complex in 3 h. The precipitates were dried over CaCl_2_ in a desiccator.

#### 2.3.1. Fe(AG‐PAA)Cl_2_(H_2_O)_2_

The Fe(III) complex data are as follows: Color = brown. Anal. Calcd. (Found) for C9H13FeO9Cl2 (MW = 391.94 g/mol), C = 27.58 (27.05) % and H = 3.34 (3.15) %. FT‐IR (KBr, cm−1) = 3200 *υ*(O‐H), 1420 *υ*(COO)sym, 1714 *υ*COO)asym, 1036 *υ*(COC), 593 *υ*(M‐O) and 418 *υ*(M‐Cl). UV–Visible (DMSO, nm) = 287 and 520. Λ (DMSO, Ω−1cm2mol−1) = 19.05. μeff (BM) = 5.92.

#### 2.3.2. Cr(AG‐PAA)Cl_2_(H_2_O)_2_

The Cr(III) complex data are as follows: Color = dark green. Anal. Calcd. (Found) for C9H13CrO9Cl2 (MW = 388.10 g/mol), C = 27.85 (27.91) % and H = 3.38 (3.59) %. FT‐IR (KBr, cm−1) = 3290 υ(O‐H), 1419 *υ*(COO)sym, 1700 *υ*(COO)asym, 1034 *υ*(COC), 559 *υ*(M‐O) and 414v *υ*(M‐Cl). UV–Visible (DMSO, nm) = 300 and 548. Λ (DMSO, Ω−1cm2mol−1) = 25.57. μeff (BM) = 3.91.

### 2.4. Biological Analysis

#### 2.4.1. Antimicrobial Activity

Antimicrobial activity was assessed through the agar diffusion technique [25]. The testing involved the bacteria *Staphylococcus aureus*, *Escherichia coli*, *Salmonella typhimurium,* and *Micrococcus luteus*. The pathogenic yeast strain *Candida albicans* for the antifungal assay was also used. Inocula of all bacteria and the yeast strain were cultured onto Muller–Hinton (MH) agar plates, before filter paper discs (6 mm in diameter, Biolife Italy) were placed on the agar surfaces. These discs were impregnated with our compounds (20 μL, 10 mg/mL in DMSO). After culturing for 3 days, the antibacterial activities were indicated by measuring the diameters of the inhibition zones. All experiments were performed in triplicate.

#### 2.4.2. DPPH Free Radical Scavenging Activity

Antioxidant activity was evaluated via the determination of the DPPH free radical scavenging potential. DPPH, as a stable synthetic free radical, is frequently used to evaluate a compound’s capacity to scavenge free radicals in ethanol and water [26]. The procedure involved mixing 2 mL of several compound solutions (ligand, Fe(III) complex, and Cr(III) complex) with 2 mL of DPPH solution (0.2 mM in 95% ethanol). After shaking the reaction mixture, it was allowed to settle at room temperature for half an hour in the dark. Using a spectrophotometer (Metash, model UV‐5200, Shanghai Xiwen Biotech. Co., Ltd, Shanghai, China), the absorbance was quickly measured at 517 nm. The following formula was used to determine DPPH’s free radical scavenging activity.

(1) DPPH scavenging activity %=1−Abs sampleAbs control×100.

#### 2.4.3. Cell Viability and Anticancer Assays

The American Type Culture Collection (ATCC) (Virginia, USA) provided the MCF‐7 human breast cancer cell line and the MCF‐10A normal human mammary epithelial cell line. Penicillin (100 IU/mL) and streptomycin (100 μg/mL) were among the antibiotics added to Dulbecco’s Modified Eagle’s Medium (DMEM), which served as the growth medium for all cell types. The cultures were kept at 37°C, 5% CO_2_, and 100% relative humidity in an incubator. With minor adjustments, the MTT‐based tetrazolium assay method was followed [27]. Cells were cultured in 96‐well plates, each containing 2 × 10^4^ cells. Following a 24‐h period, samples in different concentrations were added to the wells. After a 72‐h incubation period, the MTT assay was performed. The absorbance was read at 570 nm using an ELISA reader. Measurements are means of three replicates.

## 3. Results and Discussion

### 3.1. Synthesis and Characterization of the Complexes

**FIGURE 2:**
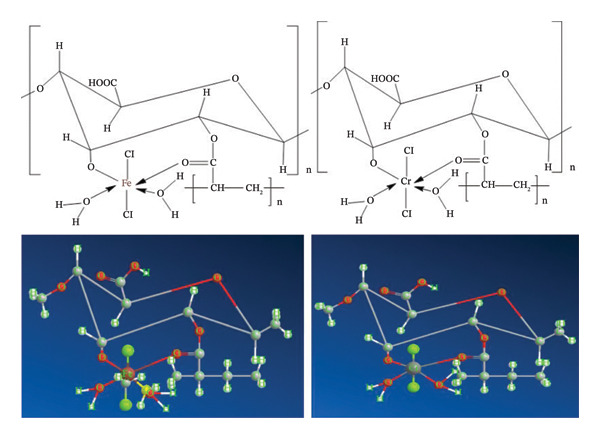
Potential structures of the Fe(AG‐PAA)Cl_2_(H_2_O)_2_ and Cr(AG‐PAA)Cl_2_(H_2_O)_2_ and a potential coordination structure around the Fe(III) and Cr(III) complexes.

Combining AG‐PAA with chlorides of Fe(III) or Cr(III) (dissolved in MeOH and distilled water) in the mole ratio of 1:1 (M:L) resulted in the creation of octahedral complex biopolymers (their possible coordination structure is shown in Figure 2). The synthesized polymeric complexes are air‐stable and insoluble in almost all common organic solvents. They showed some solubility in DMSO, which allowed us to adequately confirm their nonelectrolytic nature. The resulting biopolymers exhibit antimicrobial, antioxidant, and anticancer properties due to the synergistic effects of the metal nanoparticles and the biopolymer matrix.

**FIGURE 3:**
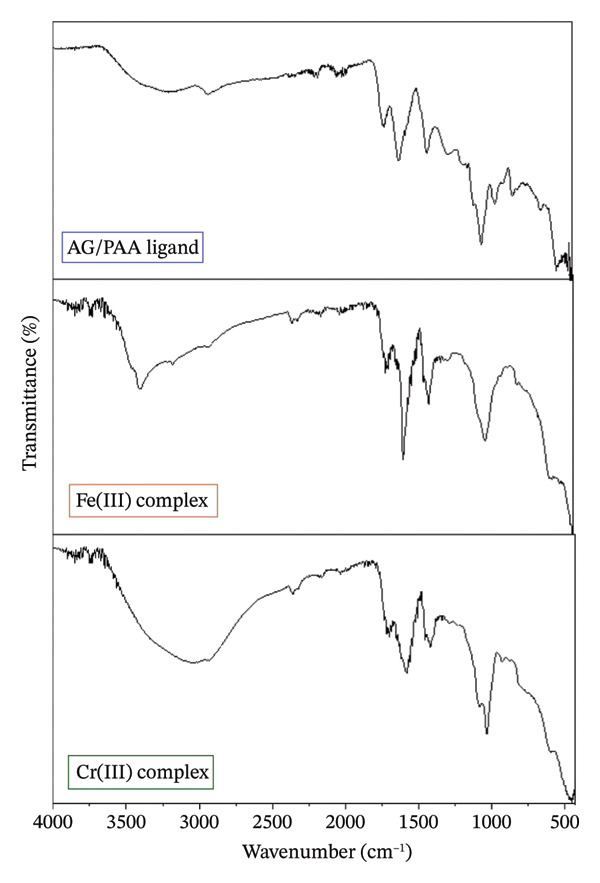
FT‐IR spectra of the ligand and its Fe(III) and Cr(III) complexes.

The functional groups in poly‐PAA/AG and the changes in wavenumber positions occurred after complexation were studied through FT‐IR spectrophotometric analysis. A collection of obtained spectra is displayed in Figure 3 and Table 1. A band at 1413 cm^−1^ identifies the ester group formation in poly‐PAA/AG due to the condensation reaction between the AG (glycosidic OH groups) and PAA (COOH groups). The FT‐IR spectrum of the complexes contains multiple characteristic bands that simultaneously appeared in the spectra of the starting biopolymer. In the ligand, a broad band exists at 3222 cm^−1^ representing hydroxyl group stretching vibration, and this band suffered a small shift on complexation [2]. The ligand glycosidic structure showed its (C‐O‐C) stretching vibration through strong absorption at 1025 cm^−1^ [28], which shifted to 1034–1036 cm^−1^ for the complexes. IR spectroscopy detected visible variations regarding both of the *υ*(COO)_sym_ and *υ*(COO)_asym_ vibrations (for the ligand, 1419 and 1725 cm^−1^, respectively). In the complexes, these bands were detected at 1420–1419 cm^−1^ and 1700–1714 cm^−1^. The separation values, Δ*υ*, between these groups in the complexes are of 281–294 cm^−1,^ indicating a monodentate mode of coordination of the carboxylate group [29]. Bands appear at 414–418 and 559–593 cm^−1^ within the IR spectra of the polymeric complexes, and these bands are due to stretching vibrations of M‐Cl and M‐O bonds, respectively [30, 31].

**TABLE 1:** FT‐IR spectral bands and their assignments of the ligand and the Fe(III), and Cr(III) complexes.

Assignment	AG‐PAA	Fe(III) complex	Cr(III) complex
^v^(O‐H)	3222	3200	3290
^v^(C‐H)	2922	2936	2935
^v^(COO) _sym_	1413	1420	1419
^v^(COO) _asym_	1725	1714	1700
υΔ	312	294	281
^v^(C=C)	1600	1585	1581
^v^(CO)	1263	1290	1294
^v^(COC)	1025	1036	1034
^v^(M‐O)	—	593	559
^v^ (M‐Cl)	—	418	414

*Note: ν*(C=O) represents the frequencies of the ester carbonyl obtained after condensation of the AG and the PAA.

The UV–Visible spectra of AG, PAA, and the MCPs were measured in DMSO. The organics did not show clear absorption maxima, although some weak peaks occurred that cannot be perfectly distinguished due to their locations in the solvent cutoff region. For the complexes, these peaks were noticed at 287 nm (for the Fe(III) complex) and 300 nm (for the Cr(III) complex). Also, the complexes showed peaks at 520 nm (Fe(III) complex, ^6^A_1g_ ⟶ ^4^T_2g_ (G)) and 548 nm (Cr(III) complex, ^4^A_2g_ ⟶ ^4^T_2g_ (F)), indicating their octahedral geometry [8, 32–34]. The octahedral geometry is also supported by the magnetic moment values of the complexes (5.92 and 3.91 BM for high‐spin iron(III) and chromium(III) complexes, respectively) [30, 33, 35].

**FIGURE 4:**
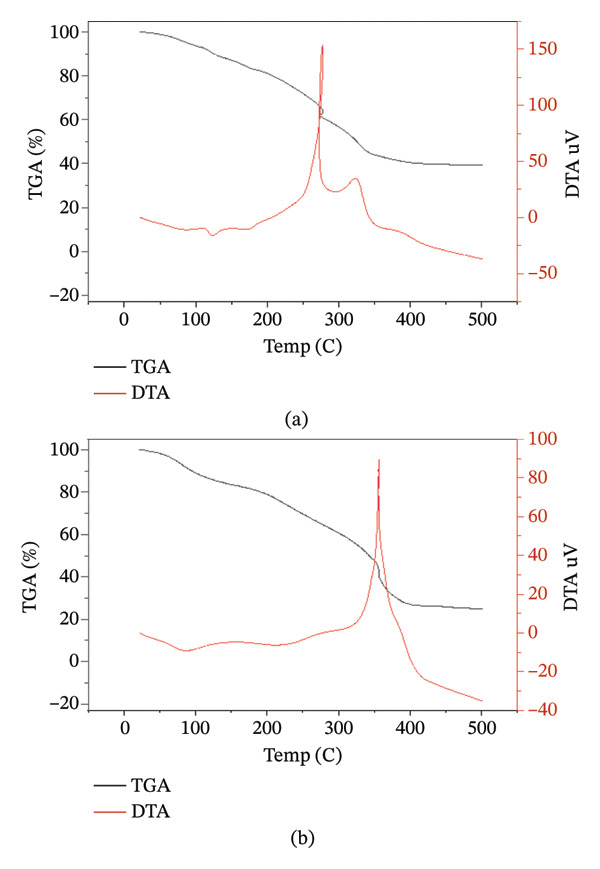
TGA and DTA thermograms of the Fe(III), above, and Cr(III), below, complexes.

Thermal analysis: The thermogram for the Fe(III) complex’s thermal behavior (Figure 4,) shows that there are four distinct steps of mass loss: 66°C–127°C, 129°C–142°C, 144°C–350°C, and 352°C–500°C. The first step is consistent with the release of the 2H_2_O molecules (calcd. 9.19%, obs. 9.33%) with a DTG midpoint at 75°C, which is associated with an endothermic peak at 77°C in the DTA trace. Two chloride atoms are expelled, as indicated by the mass loss seen in the second step (calcd. 18.08%, obs. 18.20%). According to the third and fourth mass losses (calculated 58.71%, found 58.10%), the remaining organic (AG‐PAA) ligands are released between 144°C and 350°C. Between 352°C and 500°C, the final product (calculated 18.32%, found 18.74%) agrees with 1/2Fe_2_O_3_.

Four stages of weight loss are displayed on the Cr(III) complex’s TG curve. The first stage of decomposition, in which the loss of mass is about 9.35% (calc. 9.28%), corresponds to the loss of 2H_2_O molecules and happens between 68°C and 132°C. As a result of the second stage of the decomposition process, two chloride atoms are lost at temperatures between 134°C and 245°C, resulting in a mass loss of 17.73% (calc. 18.26%). The remaining organic (AG‐PAA) ligands are released as indicated by the third and fourth mass losses (calc. 59.29%, found 55.72%) and happen between 247°C and 325°C. The final product (calculated at 19.57%, found at 19.10%) agrees with 1/2Cr_2_O_3_ (Figure 4, below).

**FIGURE 5:**
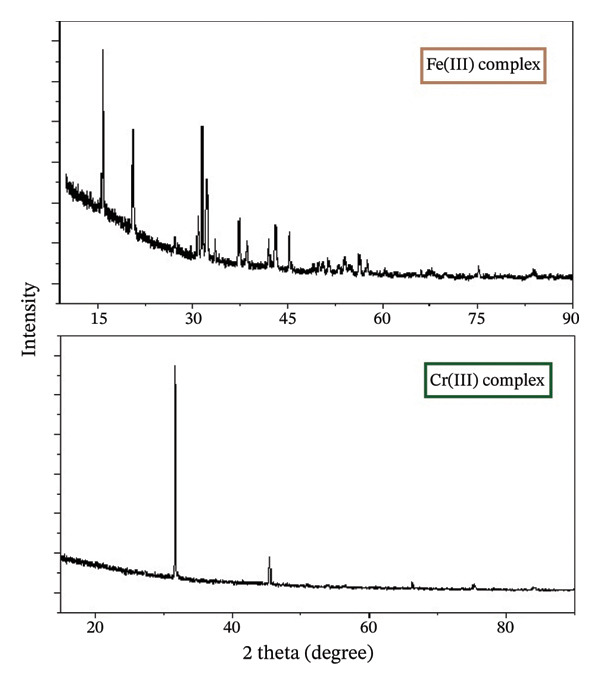
XRD patterns of the Fe(III) and Cr(III) complexes.

X‐Ray diffraction (XRD) is a powerful analytical technique used to determine the crystallographic structure, phase composition, and other properties of materials. Table 2 lists selected crystallographic parameters for the complexes. This analysis investigated the amorphous structure of the ligand, unlike the coordination compounds, which are crystalline (Figure 5). Also, the analysis assigned cubic crystal structures for the complexes and, based on Scherrer’s equation, their nanoscaled structures (35–40 nm for the most prominent peaks) were shown.

**TABLE 2:** X‐Ray powder diffraction data of the Fe(III) and Cr(III) complexes (Cu‐Kα radiation [λ_Cu_ = 0.154,059 Å]).

Parameters	Fe (III) complex	Cr(III) complex
Empirical formula	C_9_H_13_FeO_9_Cl_2_	C_9_H_13_CrO_9_Cl_2_
Formula weight	391.94	388.10
Crystal system	Cubic	Cubic
a (Å)	3.9916	3.9872
b (Å)	3.9916	3.9872
c (Å)	3.9916	3.9872
Alpha (°)	90.00	90.00
Beta (°)	90.00	90.00
Gamma (°)	90.00	90.00
Volume of unit cell (Å^3^)	63.596	63.39
Particle size (nm)	40	35
Space group	Pm‐3m	Pm‐3m

**FIGURE 6:**
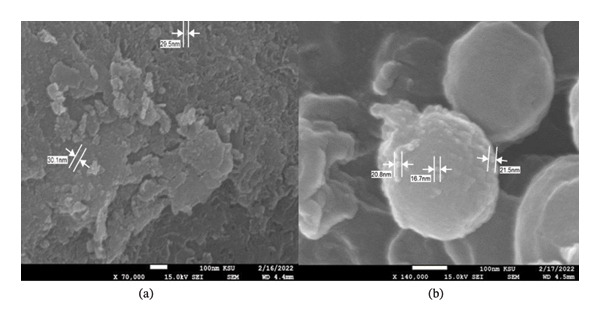
SEM images of the (a) Fe(III) and (b) Cr(III) complexes.

**SEM** detected the structures of the complexes at the microscopic levels (Figure 6) [36]. The porous and irregular surface of the cross‐linked polymeric ligand (poly‐PAA/AG) was significantly hydrophilic. The images of the Fe(III) compound’s surface showed various‐shaped particles with diameters ranging from 30 to 40 nm, but the Cr(III) complex’s SEM images showed a surface of spherical particles with diameters of 15–20 nm. The variation in particle morphology is likely related to the different coordination behavior, ionic radii, and complexation kinetics of the two metal ions. Fe(III), owing to its higher tendency toward rapid hydrolysis and variable coordination interactions, may promote nonuniform nucleation and irregular aggregation, resulting in a less regular morphology. In contrast, Cr(III) generally exhibits slower ligand exchange kinetics and more stable coordination behavior, which can favor controlled growth and the formation of more uniform spherical particles. In addition, differences in metal–ligand cross‐linking density may influence the packing and growth process of the polymeric network.

### 3.2. Biological Assessment of the Prepared Polymeric Complexes

#### 3.2.1. Antimicrobial and Antioxidant Assays

We investigated the antimicrobial activities against Gram‐negative and Gram‐positive bacteria (*Staphylococcus aureus*, *Micrococcus luteus*, *Escherichia coli*, and *Salmonella typhimurium*) as well as the yeast strain (*Candida albicans*) via the agar diffusion technique. As seen in Table 3, more effectiveness was displayed by the iron complex, which is noticeably always more active than the ligand. The highest activity was determined by the iron(III) complex against *S. aureus* (14.5 mm) and by the chromium complex against *Micrococcus luteus* (15 mm). This detected antimicrobial activity, which matches findings from previous research on AG and carboxylic acid compounds [37], is probably due to disruption of the bacterial membranes by the metals, after controlled release of them at reaction sites [38]. The absence of activity of the Cr(III) complex against Gram‐negative bacteria and yeast may be attributed to its higher coordination stability and lower redox activity, which can reduce its interaction with microbial cell components and limit reactive oxygen species generation. In contrast, the Fe(III) complex may exhibit enhanced biological activity due to its redox behavior and greater ability to induce oxidative stress, leading to membrane damage and disruption of cellular processes. Furthermore, the outer membrane of Gram‐negative bacteria acts as an additional permeability barrier, which may further restrict the uptake of the Cr(III) complex. Regarding the antioxidant action (DPPH scavenging activity), all ligand and complex polymers offered high antioxidant potential, with the iron complex offering the highest, followed by the ligand.

**TABLE 3:** The antimicrobial (mm) and antioxidant (%) potential of the ligand and complexes.

**Antimicrobial activity (inhibition zone [mm])**						**Antioxidant (%)**
	***Staphylococcus aureus***	***Micrococcus luteus***	***Escherichia coli***	***Salmonella typhimurium***	***Candida albicans***	

Ligand	9	8	9.2	10.4	0	93^±1.4^
Fe(III) complex	14.5	12	13	12.5	11.5	95.5^±0.4^
Cr(III) complex	12	15	0	0	0	60.5^±0.5^

#### 3.2.2. In Vitro Anticancer Evaluation

**FIGURE 7:**
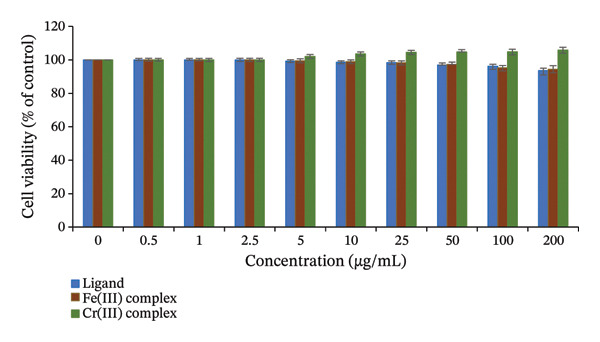
Biocompatibility of the ligand and the two complexes via the cell viability assays with human normal breast cells (MCF‐7) was evaluated after incubation for 72 h at increased concentrations.

**FIGURE 8:**
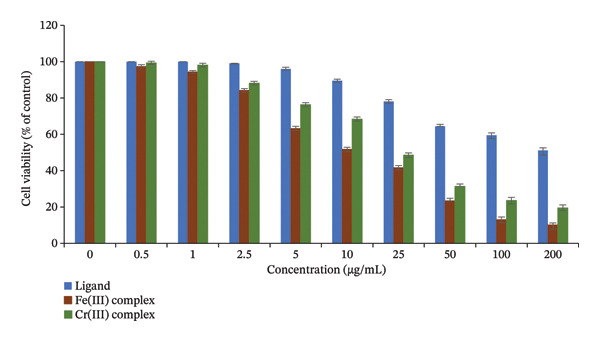
Anticancer activity of the ligand and the two complexes with breast cancer cell lines (MCF‐10A) was evaluated after incubation for 72 h at increased concentrations.

A laboratory MTT examination of the synthesized ligand polymer and complexes as anticancer agents against MCF‐7 human breast cancer cells, as well as normal epithelial cells (MCF‐10A), was performed. The ligand remained nontoxic toward the normal cells, aligning with several previously published studies [37]. This confirms the excellent biocompatibility of naturally derived polysaccharides and assumes safe usage of the cross‐linked polymer of AG and PAA for drug delivery applications. The normal human breast epithelial cells were also not influenced by the complexes. This is a significant finding because drugs must selectively target cancer cells. Regarding the action against MCF‐7 cancer cells, the ligand killed these cells with an IC_50_ of 236.5 μM. In a dose‐dependent manner, the complexes exhibited significant cancer cell–killing ability with IC_50_ values of 11.8 μM (for the iron complex) and 20.6 μM (for the chromium complex). It should be noted that, after 72 h of incubation, the highest tested concentration, “200 μg/mL,” reduced the viability inhibition of these cancer cells to 10.2 ± 1.12% (the Fe(III) complex) and 19.6 ± 1.22% (the Cr(III) complex) (44% viability inhibition by the ligand). This in vitro anticancer evaluation showed that the produced polymer compounds had strong anticancer activity, which was notably enhanced at higher doses. The AG’s biocompatibility and the Fe(III)’s ability to induce oxidative damage make their complex an attractive candidate for anticancer drug delivery. The mechanism of action can be due to the generation of ROS to damage the cancer cells, while the AG/PAA matrix can protect the iron from premature release until it reaches the tumor site, allowing for targeted therapy [38, 39] (Figures 7 and 8).

## 4. Conclusion

Synthesized cross‐linked AG/PAA biopolymer functions exceptionally well as a binding agent for Fe(III) and Cr(III) metal cations. Coordination polymers demonstrated strong antimicrobial functions through their antibacterial and antifungal properties. Tests documented that the created iron(III) and Cr(III) coordination polymer solutions successfully inhibited Gram‐positive and Gram‐negative bacteria types. The synthesized coordination polymers, together with the polymeric ligand, expressed robust antioxidant chromosomal behavior. Anticancer potency of these complexes against human breast cancer cells (MCF‐7) increased with the administered dose. The IC50 value identifying the anticancer activity of Fe(III) coordination polymer reached 11.8 μM at 10.52 μg/mL, and the Cr(III) coordination polymer reached 20.6 μM at 19.3 μg/mL.

## Author Contributions

Data curation, Maged S. Al‐Fakeh, Yassine El‐Ghoul, and Maged S. Al‐Fakeh; formal analysis: Maged S. Al‐Fakeh, Yassine El‐Ghoul, and Maged S. Al‐Fakeh; investigation: Maged S. Al‐Fakeh, Yassine El‐Ghoul, and Maged S. Al‐Fakeh; methodology: Maged S. Al‐Fakeh, Yassine El‐Ghoul, and Maged S. Al‐Fakeh; administration: Maged S. Al‐Fakeh; software: Maged S. Al‐Fakeh, Yassine El‐Ghoul, Maryam Aldoghaim, and Maged S. Al‐Fakeh; supervision: Maged S. Al‐Fakeh and Yassine El‐Ghoul; validation: Maged S. Al‐Fakeh, Yassine El‐Ghoul, Munirah S. Alazmi, and Ahmed B. M. Ibrahim; writing–original draft: Maged S. Al‐Fakeh, Yassine El‐Ghoul, Maryam Aldoghaim, Maged S. Al‐Fakeh, and Ahmed B. M. Ibrahim; writing–review and editing: Maged S. Al‐Fakeh, Maryam Aldoghaim, Yassine El‐Ghoul, Munirah S. Alazmi, and Ahmed B. M. Ibrahim.

## Funding

This study was supported by the Deanship of Scientific Research, Vice Presidency for Graduate Studies and Scientific Research, King Faisal University, Saudi Arabia (Grant no. KFU250165).

## Disclosure

All authors have read and agreed to the published version of the manuscript.

## Conflicts of Interest

The authors declare no conflicts of interest.

## Data Availability

The data that support the findings of this study are available from the corresponding authors upon reasonable request.
